# New dating evidence of the early presence of hominins in Southern Europe

**DOI:** 10.1038/s41598-017-10178-4

**Published:** 2017-08-30

**Authors:** Véronique Michel, Chuan-Chou Shen, Jon Woodhead, Hsun-Ming Hu, Chung-Che Wu, Pierre-Élie Moullé, Samir Khatib, Dominique Cauche, Marie-Hélène Moncel, Patricia Valensi, Yu-Min Chou, Sylvain Gallet, Anna Echassoux, François Orange, Henry de Lumley

**Affiliations:** 1 Université Côte d’Azur, CNRS, CEPAM, 06357 Nice, France; 2 Université Côte d’Azur, CNRS, OCA, IRD, Géoazur, 06560 Valbonne, France; 3 High-Precision Mass Spectrometry and Environment Change Laboratory (HISPEC), Department of Geosciences, National Taiwan University, 10617 Taipei, Taiwan R.O.C.; 4 School of Earth Sciences, University of Melbourne, VIC, 3010 Australia; 5 Musée de Préhistoire Régionale de Menton, 06500 Menton, France; 6 Institut de Paléontologie Humaine, 75013 Paris, France; 7 Laboratoire Nice Côte d’Azur, 06300 Nice, France; 8 Département de Préhistoire, MNHN Paris, CNRS, 75013 Paris, France; 9 Musée de Préhistoire, 06690 Tourrette-Levens, France; 10 Réserve de biosphère de Fontainebleau et du Gâtinais, 77300 Fontainebleau, France; 11 Université Côte d’Azur, Centre Commun de Microscopie Appliquée (CCMA), 06108 Nice, France

## Abstract

The first “Out of Africa” migrations represent a seminal event in the history of humankind. At the gates of Europe, the first appearance of Hominins is recorded in Georgia, 1.8 million years ago (Ma); however, the picture of migration across the continent remains incomplete. Vallonnet Cave (France) is a Lower Paleolithic prehistoric site with traces of hominin activities including lithic remains and cut-marks on mammal bones. Here, we apply the uranium-lead (U-Pb) methods to two flowstones to date the intervening archaeological levels. The U-Pb data, coupled with paleomagnetic constraints, provide an age range from 1.2 to 1.1 Ma. The results conclusively demonstrate that Vallonnet Cave is one of the oldest European prehistoric sites in France with early hominin occupations associated with an Epivillafranchian fauna. Combined with data from other archaeological sites, the new precise chronology suggests a widespread occupation the Northern Mediterranean to Southwestern Europe at ~1.2 Ma.

## Introduction

**Figure 1:**
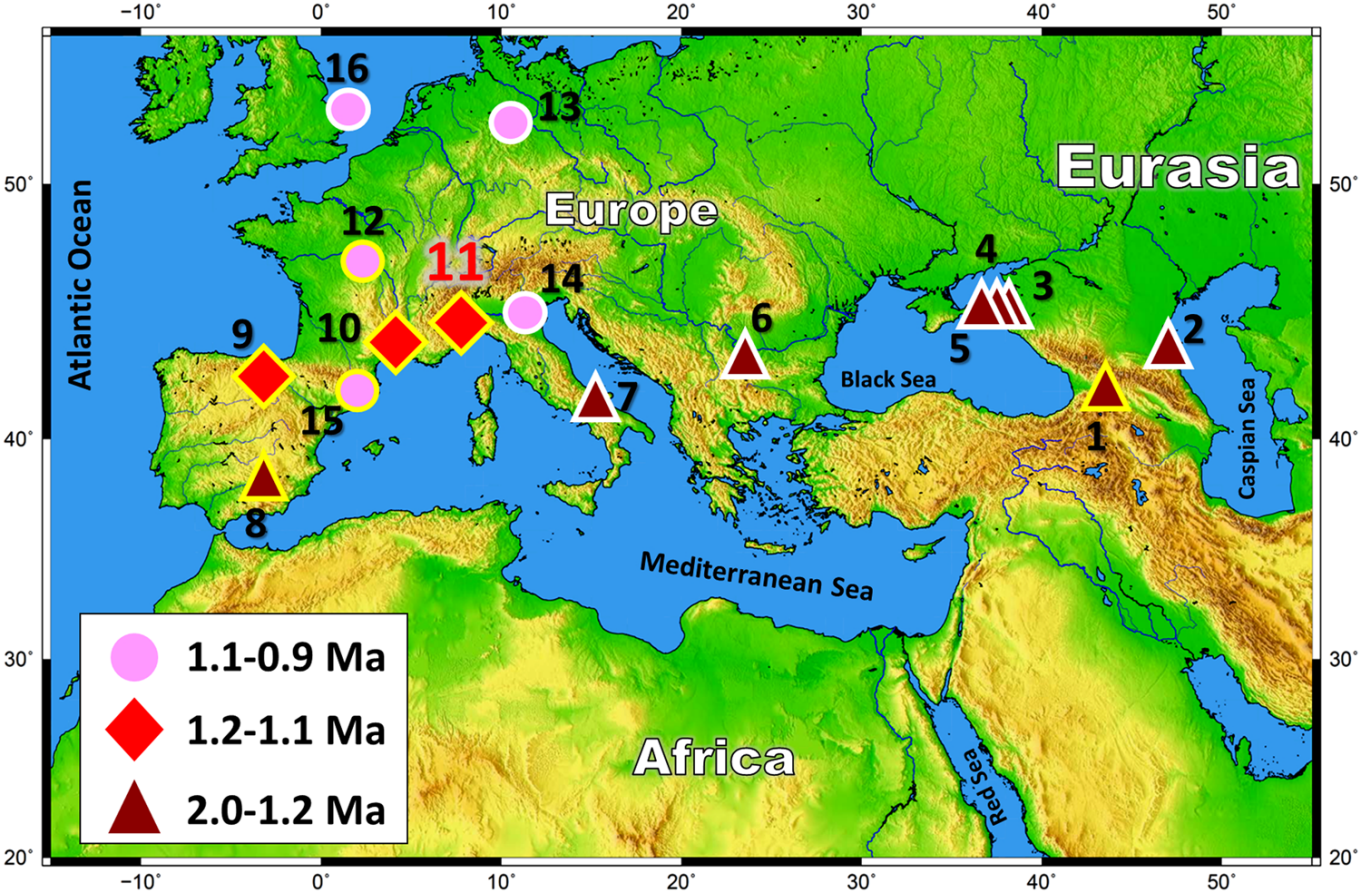
The first “Out of Africa” migrations toward Eurasia and Europe. Geographical distribution of Pleistocene sites with Oldowayen culture in the circum-Mediterranean, Western Europe region discussed in the main text. Symbols with yellow borders are the sites where multiple dating methods, including radiometric techniques, were applied. Symbols with white borders are the sites dated using biostratigraphy and/or palaeomagnetism without radiometric dating. Dark red triangles correspond to ages ranging from 2.0–1.2 Ma for sites of (**1**) Dmamisi^5, 6^, (**2**) Muhkai II^41^, (**3**) Kermek^42^, (**4**) Rodniki^42^, (**5**) Bogatyri^42^, (**6**) Kozarnika^43^, (**7**) Pirro Nord^44^, and (**8**) Orce^7, 8^; red diamonds to 1.2–1.1 Ma at (**9**) Sima del Elefante^9^, (**10**) Bois-de-Riquet^10^, (**11**) and Vallonnet (this study); and pink circles to ages around 1.1–0.9 Ma at (**12**) Lunery^13^, (**13**) Untermassfeld^14^, (**14**) Monte Poggiolo^15^, (**15**) Vallparadis^16^ and (**16**) Happisburgh^17^. This map was created with software Generic Mapping Tools (GMT) Graphics v. 5.1.1.

Our understanding of hominin evolution, hominin migration and cultural change relies fundamentally on the establishment of accurate chronological frameworks. The earliest evidence of hominin tool manufacture is dated from Kenya and in Ethiopia (Africa) *via* precise ^40^Ar/^39^Ar dating of the Lomekwi 3 (refs 1 and 2) and Gona sites^3, 4^. The first “Out of Africa” migrations toward western Europe are recorded by the presence of an increase of well-dated sites with hominin settlements such as Dmanisi (Georgia, 1.85–1.78 Ma) (refs 5 and 6), Orce (Spain, 1.5–1.4 Ma) (refs 7 and 8), La Sima del Elefante (Spain, 1.2–1.1 Ma) (ref. 9) and Bois-de-Riquet (France, 1.3–1.1 Ma) (ref. 10) (Fig. 1). From a cultural viewpoint, the earliest evidence of hominin occupation in Europe corresponding to the “Oldowayen” is contemporaneous with the earliest bifaces in East Africa around 1.8–1.7 Ma (refs 11 and 12). At ~1 Ma, Mode 1 (core-and-flake) industries continued, both in the south and north of Europe (i.e. Lunery at ~1.1 Ma in France^13^; Untermassfeld at ~1.1 Ma in Germany^14^; Monte Poggiolo at 0.9 Ma in Italy^15^; Vallparadis at 0.9 Ma in Spain^16^; Happisburgh at 0.9 Ma in Great Britain^17^) alongside the onset of the first bifaces in Europe, probably close to 0.7 Ma (refs 18–21).

In this context, existing (relatively imprecise) dating of the Vallonnet site (Supplementary Fig. 1 and Supplementary Note 1) to 1.37–0.91 Ma using the electron spin resonance (ESR) method on calcite^22^ suggests that it potentially records key events during this early phase of hominin history, such as the first hominin dispersals in Europe with Mode 1 industries (Fig. 1 and Supplementary Note 2). For more than two decades, Vallonnet Cave has been extensively cited in the literature as being synchronous with the Jaramillo paleomagnetic subchron. The ESR on calcite dating method used, however, is now controversial and has not been applied for some time. Furthermore, the equivalent dose and the annual dose rates, feeding into the age calculation, were not estimated with great precision, leading to a large range of dates with significant uncertainties, i.e., 0.91 ± 0.06 Ma at the base of the Upper flowstone and 1.37 ± 0.12 Ma at the top of the Lower flowstone. In view of the importance of this site for understanding Early Pleistocene hominin evolution in southern France, more accurate dating is required. To improve the time scale for deposition of the Vallonnet Cave infilling, a dating program was undertaken using radiometric dating techniques combined with paleomagnetism in a well-known stratigraphic context (Supplementary Fig. 2 and Supplementary Note 1).

**Figure 2:**
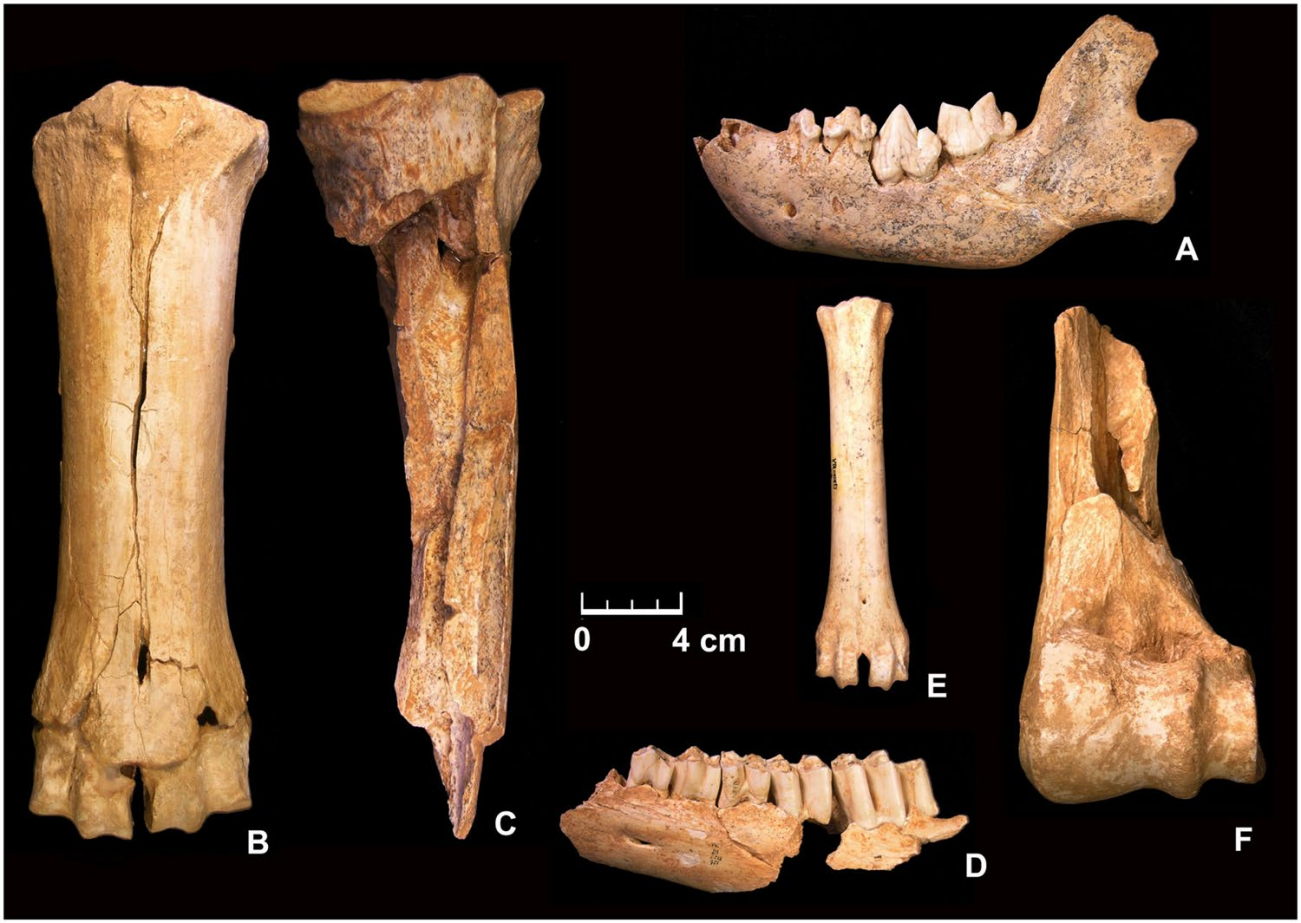
Large mammal fauna from Vallonnet Cave. (**A**) *Pachycrocuta brevirostris*, left mandible with dp2, dp3, P4, M1 in lateral view (Val-C4-CE8–149). (**B**) *Bison schoetensacki*, right metacarpal in dorsal view (Val-A7-B1–3748). (**C**) *Bison schoetensacki*, left radio-ulna with the diaphyses and the proximal extremity of radius (Val-D4-DE11-171), in dorsal-medial view, showing anthropic activities. (**D**) *Ammotragus europaeus*, left mandible with P3, P4, M1, M2, M3 (Val-B9-BJ13-751). (**E**) *Hemitragus bonali*, left metatarsal in dorsal view (Val-C9-CJ21-647). (**F**) *Praemegaceros* cf. *verticornis*, distal extremity of left humerus (Val-C8-B2-1859), in cranial view, showing anthropic activities.

**Figure 3:**
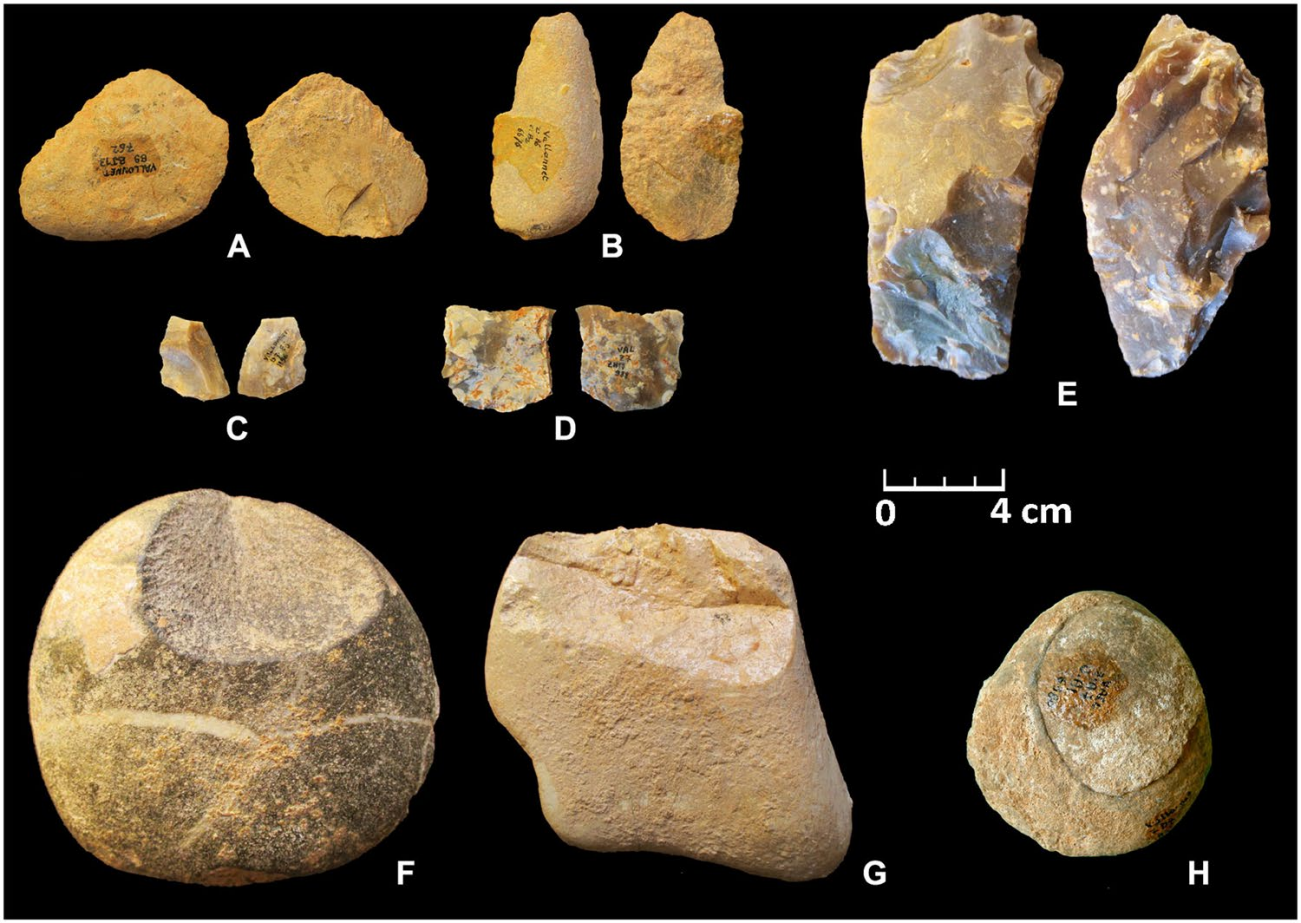
Lithic artefacts. (**A**) Flake with cortical surface and no butt, of limestone. (**B**) Flake with cortical surface and no butt, of quartzite. (**C** and **D**) Flakes of flint, from *débitage*. (**E**) Core of flint. (**F**) Pebble of quartzite, with a removal negative (percussion instrument). (**G**) Chopper on limestone flat pebble. (**H**) Refitting flake on hammerstone of sandy limestone.

Our reconnaissance studies utilized U-Th dating methods on speleothems^23^ in the hope of dating the interstratified archaeological levels, i.e., the fauna attributed to the Epivillafranchian^24, 25^ (Fig. 2, Supplementary Table 1 and Supplementary Note 3). Some cut-marks and broken bones with impact points indicate that hominins came to this cave to scavenge large and middle-sized cervid and bison carcasses, and occasionally rhinoceros, brought to the site by carnivores^26^ (Supplementary Fig. 3). The associated lithic industry includes cores, pebble-tools, flakes on local pebbles and semi-local raw materials for scavenging in the cave^27, 28^ (Fig. 3, Supplementary Fig. 4 and Supplementary Note 2). Questioned in the past^29, 30^, the 97 artifacts indicate an obvious scarce and sporadic hominin presence into the cave with *façonnage* activities and direct and bipolar evidence of debitage on limestone, flint and quartzite. Under favorable circumstances, the range of materials that can be dated with the U-Th method can extend to 800 thousand years ago using new ^234^U and ^230^Th decay constants and sub-permil (‰) isotopic measurements by Faraday cup protocols on multi-collector inductively coupled mass spectrometers (MC-ICP-MS)^31, 32^. Unfortunately, the flowstone samples proved to be beyond the limits of the U-Th dating method. For speleothems with ages beyond this, the recently developed U-Pb in the speleothem chronometer provides a viable alternative. The method was pioneered in the study of Quaternary speleothems from Winnat’s Head Cave^33^ and since that time, has been applied in a variety of contexts such as studies of landscape evolution^34^, origins of the *Homo* genus^35^, and temporal constraints on vertebrate evolution^36^. Recent progress in U-Pb dating, allowing for the dating of “young” calcite with ppb-level Pb content^37, 38^, then provided an opportunity for radiometric dating the Vallonnet Cave speleothems. U-Pb and paleomagnetism analyses were used to estimate the age of archaeological deposits at complex III.

**Figure 4:**
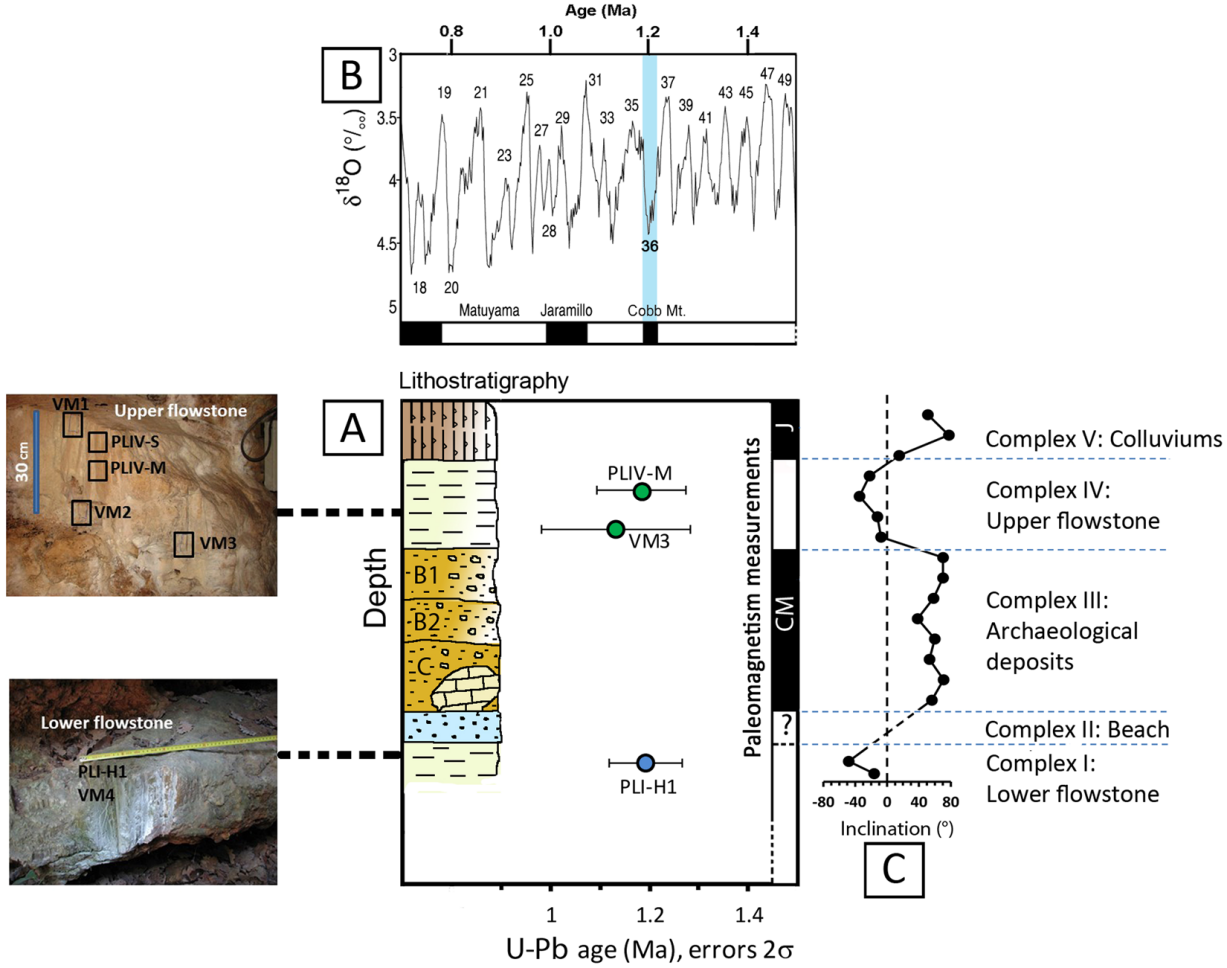
Vallonnet Cave, sampling and dating results in geological context. (**A**) Two calcite samples were taken from Lower flowstone I and five from Upper flowtone IV for U-Th content and isotopic determination and U-Pb dating (Supplementary Figs 2 and 5 and Supplementary Tables 2 and 3). The U-Pb ages of calcite samples, PLI-H1 of complex I and PLIV-M and VM3 of complex IV. “J” and “CM” respectively represent the paleomagnetic subchrons termed Jaramillo and Cobb Mountain. (**B**) The data are compared with the stacked δ^18^O record of benthic foraminifera^72^. Numbers are marine isotope stages (MIS). Vertical blue bar denotes the paleomagnetic Cobb Mountain subchron from 1.215–1.190 Ma (ref. 39) at MIS 36. (**C**) Depth profile of measured paleomagnetic inclination from complexes I–V. The archaeological deposits (complex III: B1, B2, C) are contemporaneous with MIS 36.

One stratigraphic horizon, PLI-H1 of complex I below the archaeological complex III, and three horizons VM3, PLIV-M and PLIV-S of complex IV above the archaeological complex III (Fig. 4, Supplementary Figs 2 and 5) were selected for dating. Due to relatively low uranium contents of 100 s ppb (Supplementary Table 2), these are challenging materials for U-Pb geochronology. Fortunately, low Pb contents of generally a few ppb reflect a relatively small contribution from non-radiogenic (‘common’) Pb. As a result, three of the four analyzed samples produced linear arrays in Tera-Wasserburg Concordia diagrams from which relatively precise age estimates could be obtained (see Methods).

## Results

U-Pb isotope ratios of flowstone samples are presented in Supplementary Table 3 and plotted in isochron diagrams (Supplementary Fig. 6). Sample PLIV-S (not shown) returned scattered data suggesting either multiple sources of common Pb or open system behavior and could not be dated. Using measured ^234^U/^238^U ratios^31, 32^ (2σ, Supplementary Table 2) to correct for initial disequilibrium in the U-series decay chain, the two disequilibrium-corrected U-Pb ages (2σ, Supplementary Table 3) of PLI-H1 at the top of complex I, the Lower flowstone, are: 1.19 ± 0.07 and 1.22 ± 0.09 Ma [Mean Square of the Weighted Deviations (MSWD) = 10.7]. For the Upper flowstone at complex IV, the corresponding ages of the base (VM3) are: 1.13 ± 0.15 and 1.14 ± 0.15 Ma (MSWD = 2.7) and the ages of the middle (PLIV-M) are: 1.18 ± 0.09 and 1.17 ± 0.09 Ma (MSWD = 3.3) (Fig. 4 and Supplementary Fig. 6).

The Vallonnet Cave sequence displays four paleomagnetic zones (Fig. 4 and Supplementary Table 4). The Lower and Upper flowstones (complexes I and IV) show reverse magnetic polarities. The inclination of the magnetic components varies between −7° and −48° and the declination near 223° (on average). The archaeological deposits at complex III show normal magnetic polarity. Inclination varies between 38° and 70°, which is similar to the present-day inclination of 60° measured at Nice. The colluviums in complex V also show normal magnetic polarity. The reverse magnetic polarities detected in the Lower and Upper flowstones can be attributed to the Matuyama period since the U-Pb dates from these levels give an age of 1.2–1.1 Ma. The normal polarity sequence detected in the archaeological deposits (complex III) can be correlated with the Cobb Mountain subchron dated to 1.215–1.190 Ma (ref. 39) (marine isotopic stage 36, MIS 36) (Fig. 4). The normal polarity detected in the colluviums (complex V) can be attributed to one of the Upper Matuyama magnetic subchron and excursions (Punaruu, Jaramillo, Santa Rosa, Kamikatsura) or to the Brunhes period <0.780 Ma.

## Discussion

The first hominin migration out of Africa toward Eurasia is evidenced by the well-dated ^40^Ar/^39^Ar site of Dmanisi in Caucasus at 1.85–1.78 Ma (refs 5 and 6). Often, however, for key Pleistocene sites in Europe, the ages of the fossils and tools are not based on radiometric methods and are thus correspondingly imprecise^40^. Many sites have only been approximately dated using biostratigraphy and/or dating methods with low precision, such as the site of Muhkai II in Russia with an estimated age of 2.1–1.77 Ma (ref. 41); the sites of Bogatyri and Rodniki along the Azov Sea dated to 1.6–1.2 Ma and the site of Kermek to 2.1–1.8 Ma, all constrained through biostratigraphy and paleomagnetic data^42^. An age of 1.6–1.4 Ma, based on biostratigraphy, for the Kozarnika site, in Bulgaria, also suggests a possible beginning of Western Eurasian colonization *via* this country^43^. At Pirro Nord in Italy, the earliest hominin settlement is believed to date between 1.6–1.3 Ma, based on biostratigraphy, but this chronology needs to be improved by numerical dating methods^40, 44^.

A reliable chronological framework can however be constructed from well-dated sites to highlight hominin migrations (Fig. 1). In Spain, the Late Villafranchian sites of Orce, i.e., Barranco Leon (BL-D) and Fuente Nueva-3 (FN-3)^7^, comprise the oldest evidence of hominin presence recorded so far in Western Europe. The age of the BL-D site was estimated to be close to 1.4 Ma by a combination of biochronologic, paleomagnetic data and ESR dating of quartz grains^7^. The age of FN-3 was first determined at 1.19 ± 0.21 Ma using the combined U-Th/ESR method and numerical estimates on teeth^45^ and then refined to 1.50 ± 0.31 Ma using cosmogenic nuclide dating combined with a magnetostratigraphic study of the sequence^8^. Hominin (*Homo antecessor*) presence at La Sima del Elefante (Atapuerca) has also been dated to 1.2–1.1 Ma using cosmogenic nuclides and paleomagnetism^9^. The faunal assemblage here also corresponds to the Epivillafranchian taxa as noted for Vallonnet Cave^46^. In France, the Bois-de-Riquet archaeological site at Lézignan-la-Cèbe (Hérault) is encased within a basalt flow, radiometrically dated by ^40^Ar/^39^Ar to 1.57 Ma (refs 10 and 47). This represents a maximum age, in agreement with the biochronological interpretations of the archaeological levels which suggest an age of 1.3–1.1 Ma (ref. 10).

At Vallonnet Cave, the new radiometric U-Pb analyses of the two flowstones (complexes I and IV) combined with paleomagnetism measurements of the deposit infilling provide the first robust chronological framework for the site (Fig. 4). The results show the presence of hominin activities in a den for a bivouac at ~1.2 Ma, associated with a normal paleomagnetism polarity (Cobb Mountain interval) corresponding to MIS 36, a cold glacial period. This result is consistent with the fact that the archaeological levels (complex III) record a cold climate and is supported by palynological data and faunal taxa^27, 48^. Within the 2σ uncertainty on the U-Pb dates (sample PLI-H1, Supplementary Table 3), the formation of the Lower flowstone (complex I) and the deposit of the beach (complex II) can be correlated to MIS 37, just before 1.2 Ma during a warm climate (Fig. 4).

A variety of biostratigraphic studies of Early Pleistocene sites in Europe have aimed to correlate taxa with the radiometric time-scale^49–51^; in this context the Epivillafranchian paleontological period was defined^52^, with an estimated time interval of 1.2–0.9 Ma (ref. 53). A shift in the assigned age of the large mammal fauna at Vallonnet from the previously accepted paleomagnetic Jaramillo subchron (1.07–0.99 Ma) to 1.2 Ma is easily reconciled for most of the taxa (Supplementary Table 1). A biochronological comparison of the Vallonnet fauna^25^ with the fauna from Fuenta Nueva-3 (ref. 54), observed the presence of Caprini *Ammotragus europaeus* ^55^ at both sites, and noted species differences for *Bison* and *Hemitragus*: a slender *Bison* sp. and *Hemitragus* cf. *albus* for Fuente Nueva-3 (ref. 56) and *Bison schoetensacki* and *Hemitragus bonali* for Vallonnet. Because of this difference, Vallonnet was considered to be slightly younger than Fuente Nueva-3 (ref. 57). This suggestion remains valid for the new radiometric dating of Vallonnet presented in this study and the recently published chronological data from Fuente Nueva-3 pointing to an age more than 1.2 Ma for this site (i.e. ~1.5 Ma, ref. 8).

Based on Italian sites, the Vallonnet fauna appears to be more recent than the Pirro Nord faunal units (FU) and may correspond to the Colle Curti FU^25^. It is commonly admitted that Colle Curti, Vallonnet and also Untermassfeld occupy the same position on the chronological scale^50, 51^. Our new radiometric dating of Vallonnet does not negate the biostratigraphic position of the Colle Curti FU. The Pirro Nord FU (~1.4 Ma) contains *Bison degiuli* and *Praemegaceros obscurus*. The Colle Curti FU, assigned to the Jaramillo paleomagnetic interval^58^, includes *Bison* aff. *B. schoetensacki* and *Praemegaceros verticornis* ^59, 60^. With *Bison schoetensacki* and *Praemegaceros* cf. *verticornis*, the Vallonnet site can be dated to 1.2 Ma. Moreover, note that the positioning of sites such as Colle Curti and Untermassfeld^61^ during the Jaramillo paleomagnetic interval may be revised in the future with new developments in radiometric dating, as was the case in this study.

The micromammal faunal association from Vallonnet, in particular rodent species and their evolution stage^62^, is also consistent with an age of ~1.2 Ma (see Supplementary Note 3).

The new radiometric dates of ~1.2 Ma show that Vallonnet Cave is certainly the oldest site with hominin activities and Epivillafranchian fauna in southern France on the Mediterranean coast during the glacial MIS 36 (Fig. 4), with a cool and dry climate at the base of the archaeological sequence followed by temperate and humid climatic conditions. Lithic remains and cut-marks on mammal bones clearly show that hominins were present in this area (Fig. 3, Supplementary Figs 3 and 4). It thus greatly improves our knowledge of the first dispersals of the *Homo* genus “Out of Africa” during the Early Pleistocene (Calabrian) in this area of Europe. The new chronological framework is contemporaneous with Spanish sites such as La Sima del Elefante (Level TE9c) and with Bois-de-Riquet in France, suggesting a widespread synchronous Hominin activity around the Northern Mediterranean and Southern Europe at ~1.2 Ma, followed by a northward colonization at ~1.0 Ma (Fig. 1). While it remains a challenge to precisely date all archaeological sites with adequate precision, the application of robust radiometric dating techniques to current and future sites will offer further insights and understanding into the routes of Hominin dispersal of Africa in to Europe.

## Methods

### U-Th analysis

Seven samples from two flowstones of well-crystallized calcite (Supplementary Figs 2 and 5) were collected. Samples, VM1, PLIV-S, PLI-M, VM2 and VM3, were taken from the Upper flowstone IV. Samples, PLI-H1 and VM4, were collected from the Lower flowstone I (Fig. 4). The selected bulk subsamples were physically cleaned with ultrasonic methods^63^. U-Th chemistry was conducted in a class-10,000 metal-free clean room with class-100 benches at the High-precision Mass Spectrometry and Environment Change Laboratory (HISPEC), Department of Geosciences, National Taiwan University^63–65^. Uranium and thorium isotopic and contents were determined on a MC-ICP-MS, Thermo Fisher NEPTUNE, with a dry introduction system, Cetac ARIDUS^31^. Uncertainties of U-Th data were calculated^66^ at 2σ level and included corrections for procedure blanks, multiplier dark noise, abundance sensitivity, mass discrimination, and the occurrence of isotopes of interest in spike solution. Duplicate analysis was done for all samples (Supplementary Table 2).

### U-Pb dating

Two samples, PLIV-M and VM3, and one sample of PLI-H1 were collected from the Upper flowstone IV and the Lower flowstone I respectively for U-Pb dating (Supplementary Figs 2 and 5). The analytical methods followed closely those published previously following refs 37 and 67. Multiple aliquots, typically weighing ~50 mg, were removed from each flowstone sample. These calcite fragments were placed into pre-cleaned disposable polyethylene cups and moved to a multiple-HEPA filtered clean room environment. Samples were briefly leached 2 times in ultra-pure 0.01 N HCl, with each cycle lasting around a minute, and then repeatedly washed in ultra-pure water before being dried in a HEPA filtered laminar flow hood. This step is critical to the elimination of Pb contaminants resulting from sample handling which can easily dominate the Pb budget of the entire sample unless removed. Individual samples were weighed into pre-cleaned Teflon beakers and treated with sufficient 6 N HCl to ensure complete dissolution. A mixed ^233^U-^205^Pb tracer, calibrated against EarthTime (http://www.earth-time.org) reference solutions, was then weighed into the vials and each one sealed and refluxed on the hotplate for several hours to ensure complete sample-spike equilibration. Samples were then dried down and taken up in 0.6 N HBr for Pb separation using AG 1X-8 anion exchange resin. The eluate was subsequently processed through the same column filled with Eichrom TRU ion-specific resin, to separate U. Isotope ratios were determined on a Nu Plasma MC-ICP-MS using a DSN-100 desolvation unit and MicroMist glass nebulizer, operating with an uptake rate of 50–100 µl/min. Instrumental mass bias effects were monitored and corrected using NIST SRM 981 reference material in the case of Pb, and the sample’s internal ^238^U/^235^U ratio in the case of U. Instrument data files were processed initially using an in-house designed importer, operating within the Iolite environment^68^, which considers all data and reference material analyses obtained throughout a particular analytical session and permits a variety of corrections for instrumental mass bias and drift. The resulting data, corrected for instrumental effects, were then blank corrected and isotope-dilution calculations performed using the Schmitz and Schoene’s software^69^. Using these data, isochrons were plotted in the ‘Tera-Wasserburg’ isochron construction using the well-known ‘Isoplot’ software^70^. Unfortunately, this does not allow calculation of U-Pb ages corrected for initial disequilibrium in the U-series decay chains; as a result, corrected ages were calculated using in-house software, assuming negligible initial ^230^Th and ^234^U/^238^U ratios measured previously (Supplementary Fig. 6). Isotope ratio data are given in Supplementary Table 3. All samples produced slightly elevated MSWD values suggesting some scatter beyond that expected from analytical uncertainties alone, and potentially indicating some variation in ‘common’ Pb compositions inherited by the samples at their formation. However, all MSWD values are relatively low (2.7, 3.3, and 10.7) providing confidence in the age interpretation.

### Paleomagnetism

The paleomagnetic method applied in this study is the same as that applied in the previous study^71^ with modifications. Paleomagnetic sampling was performed along the whole sedimentary deposit in Vallonnet Cave (Fig. 4, Supplementary Table 4). These samples are continuous vertical cores, 7–20 cm long and 7 cm wide and thick, oriented with a compass. The flowstones were sampled with an angle grinder and then cut into cubes of 2 cm. In the archaeological deposits (gravel and sand), sampling was carried out by hand and consolidated with plaster tape. In the laboratory, these samples were consolidated with sodium silicates to obtain cubes of 2 cm. The direction of the characteristic remanent magnetization (ChRM) was retrieved by means of stepwise thermal demagnetization of up to 400 °C and alternating field (AF) of up to 60 or 80 mT. The paleomagnetic measurements were taken using a 2 G DC-Squid Superconducting rock magnetometer (SRM) at CEREGE in Aix-en-Provence, France. The raw NRM intensity values of the lower and upper flowstones (complexes I, IV) range between 1 and 14 × 10^−8^ A/m. The maximum values occur in the archaeological deposits (complex III) and in the colluviums (complex V) and range between 1 and 6 × 10^−6^ A/m.

## Electronic supplementary material

Supplementary Information
