# Supplementary material for: New dating evidence of the early presence of hominins in Southern Europe
Source: Sci Rep. 2017 Aug 30;7:10074. doi: 10.1038/s41598-017-10178-4 (PMC5577296; doi:10.1038/s41598-017-10178-4)
Supplement: Supplementary file 1 — Supplementary Information [file 41598_2017_10178_MOESM1_ESM.pdf]

Supplementary Information

New dating evidence of the early presence of hominins in Southern Europe

Véronique Michel[1,2,*], Chuan-Chou Shen[3,*], Jon Woodhead[4], Hsun-Ming Hu[3], Chung-Che Wu[3], Pierre-Élie Moullé[5], Samir Khatib[6], Dominique Cauche[6,7,8], Marie-Hélène Moncel[8,*], Patricia Valensi[8,9], Yu-Min Chou[3], Sylvain Gallet[2], Anna Echassoux[10], François Orange[11] & Henry de Lumley[6]

[1]Université Côte d'Azur, CNRS, CEPAM, 06357 Nice, France. [2]Université Côte d'Azur, CNRS, OCA, IRD, Géoazur, 06560 Valbonne, France. [3]High-Precision Mass Spectrometry and Environment Change Laboratory (HISPEC), Department of Geosciences, National Taiwan University, 10617 Taipei, Taiwan ROC. [4]School of Earth Sciences, University of Melbourne, VIC 3010, Australia. [5]Musée de Préhistoire Régionale de Menton, 06500 Menton, France. [6]Institut de Paléontologie Humaine, 75013 Paris, France. [7]Laboratoire Nice Côte d'Azur, 06300 Nice, France. [8]Département de Préhistoire, MNHN Paris, CNRS, 75013 Paris, France. [9]Musée de Préhistoire, 06690 Tourrette-Levens, France. [10]Réserve de biosphère de Fontainebleau et du Gâtinais, 77300 Fontainebleau, France. [11]Université Côte d'Azur, Centre Commun de Microscopie Appliquée (CCMA), 06108 Nice, France.

Correspondence and requests for materials should be addressed to C.-C.S. (email: river@ntu.edu.tw), V.M. (email: veronique.michel@cepam.cnrs.fr), or M.-H. Moncel (moncel@mnhn.fr).

This PDF file includes:

Supplementary Figures 1-6 p. 2-7

Supplementary Tables 1-4 p. 8-11

Supplementary Note 1. Stratigraphy and paleoenvironment p. 12

Supplementary Note 2. The lithic assemblage p. 13

Supplementary Note 3. Fauna, biostratigraphy and taphonomy p. 14

Supplementary References p. 15-17

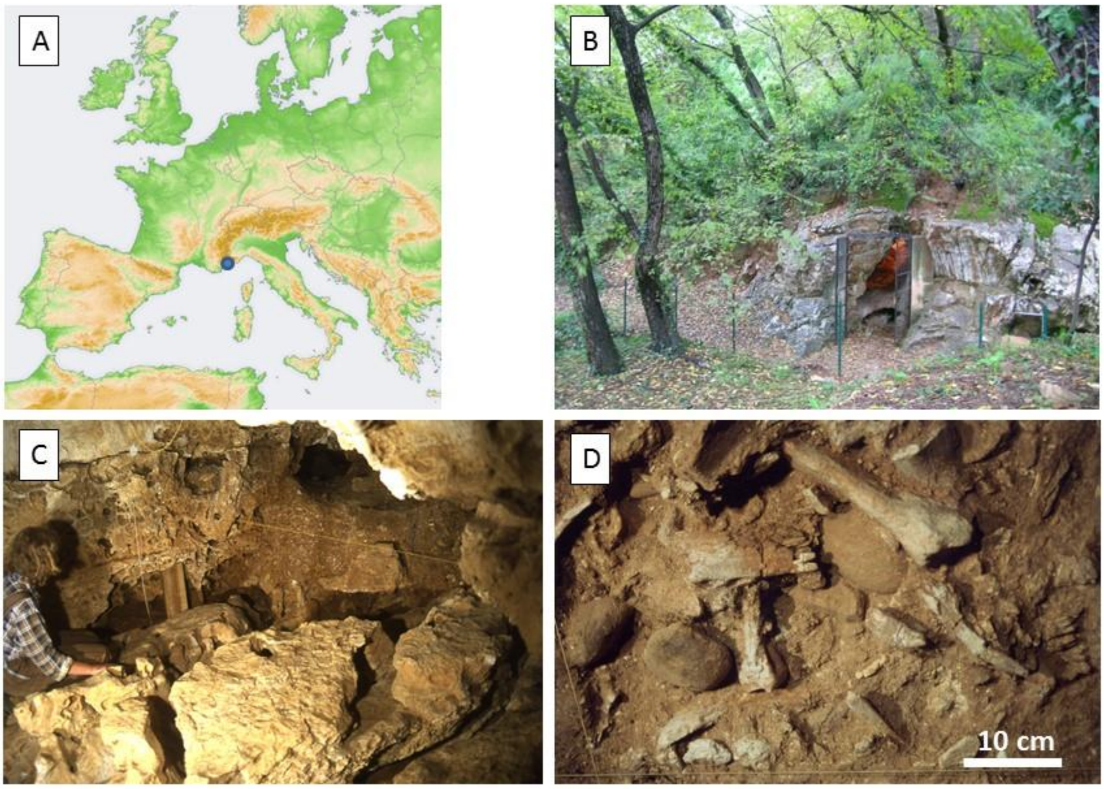

Supplementary Fig. 1. The site of Vallonnet Cave, Roquebrune-Cap-Martin, Alpes-Maritimes, France, 110 m above sea level. (A) Location in Europe (43°45’51”N 7°28’15”E). This map was modified from https://fr.wikipedia.org/wiki/Portail:Europe/Carte. **(B)** Cave entrance: width and height of 1.1 x 2.7 m. **(C)** Excavation zones 6-9 in the cave. **(D)** Complex III, excavation square A6 in zone 6, archaeological deposits with faunal remains and lithic tools.

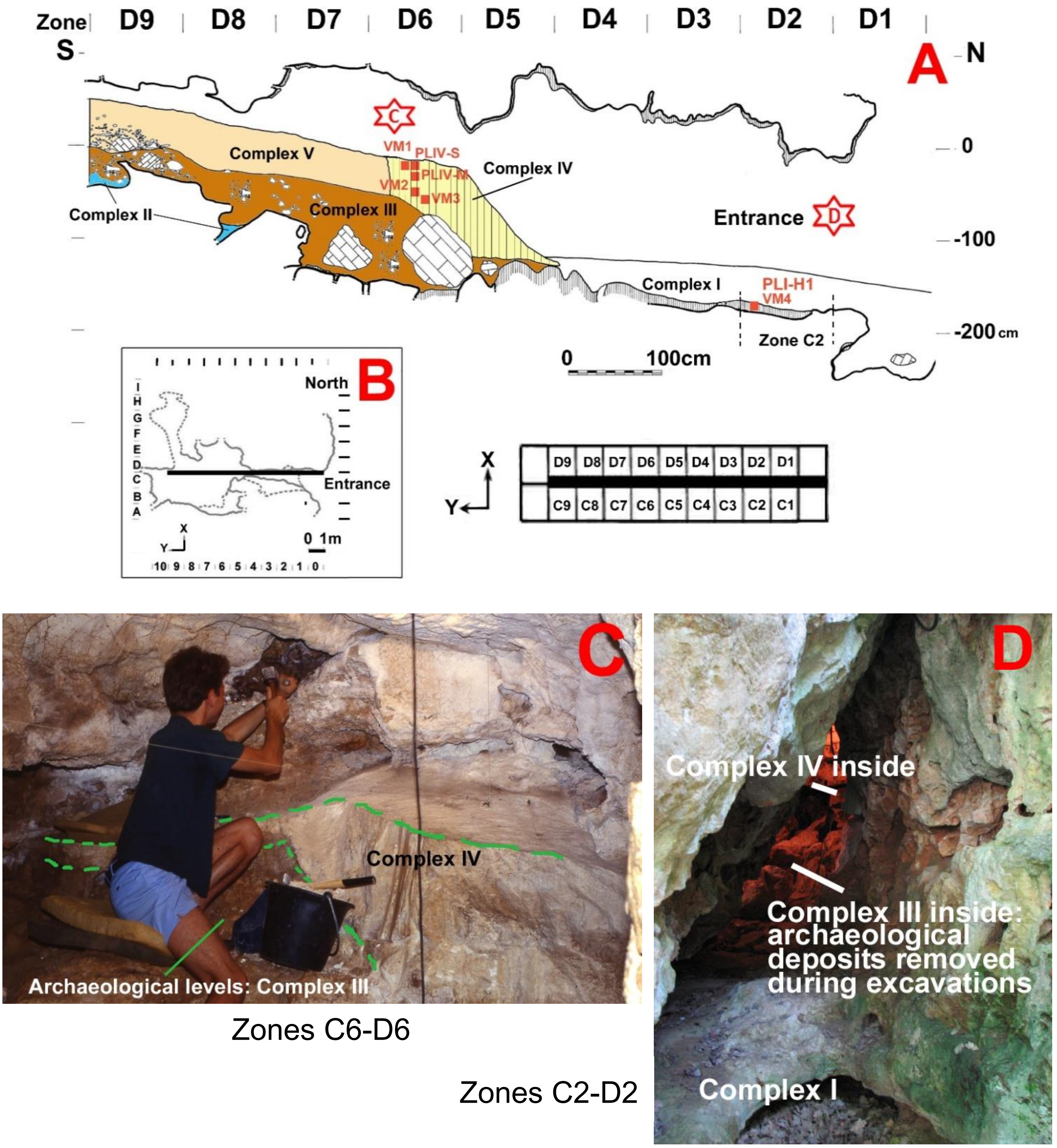

Supplementary Fig. 2. Vallonnet Cave stratigraphy. **(A)** Schematic representation of longitudinal stratigraphy section of Vallonnet Cave, with samples for dating (Zones D6 and C6, red squares), from the plan site **(B)**. The infilling sequence formed as follow (also see Supplementary Note 1, Stratigraphy and paleoenvironment). (1) Complex I is the earliest Lower flowstone. (2) A beach deposit corresponds to Complex II. (3) Archaeological levels deposits are sands, clays and silts with faunal and lithic remains as Complex III. (4) Complex IV is the Upper flowstone, it is deposited above the Complex III and sealed the corridor of cave entrance in zone D5. The lower part of the flowstone was removed to access the archaeological infilling for excavations since 1959 (ref. 73). (5) Complex V contains colluviums made up of pebbles in a yellowish-red sandy matrix. **(C)** One photograph of an excavation was taken in 1992 in Zones C6-D6 (point C in panel A), where it appears complex IV (Figure 4 in main text) above archaeological levels. **(D)** One photograph was taken at the entrance of the cave (point D in panel A), during sampling in 2013, in Zones C2-D2.

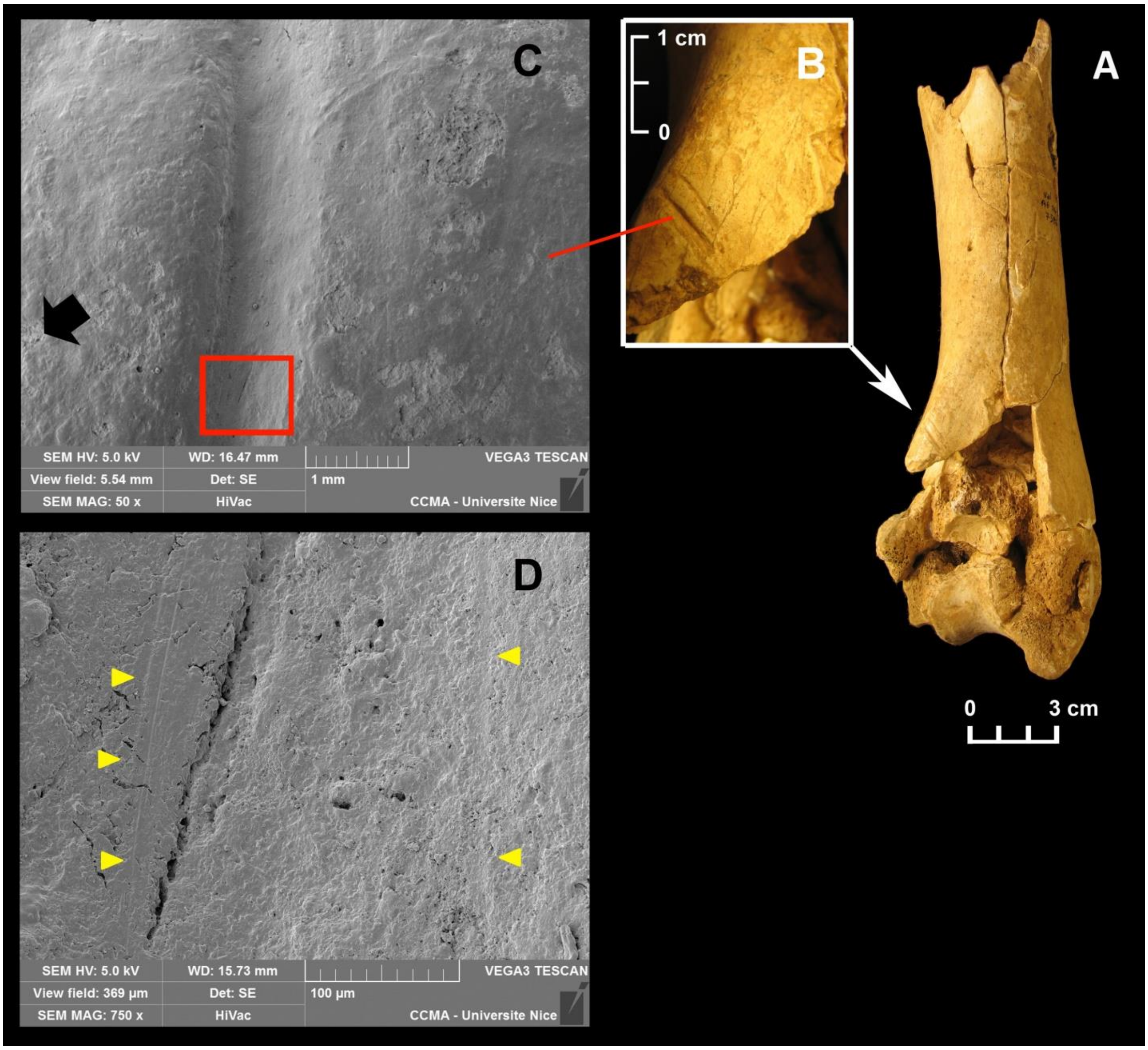

Supplementary Fig. 3. *Praemegaceros* cf. *verticornis* of Vallonnet Cave. (A) diaphysis with distal extremity of left humerus (Val-A6-AG9-7586), in caudal view, with cut-marks **(B), (C), (D)** on the lateral epicondylar crest. **(B)** Photograph of cut-marks. **(C)** SEM microphotograph of the deepest cut-mark. Striation has a well defined V-shaped cross-section. Black arrow indicates the proximal extremity of the bone. **(D)** Detail of the cut-mark (from red square in **C**) with internal straight microstriations (yellow arrows) arising from the use of edge tool.

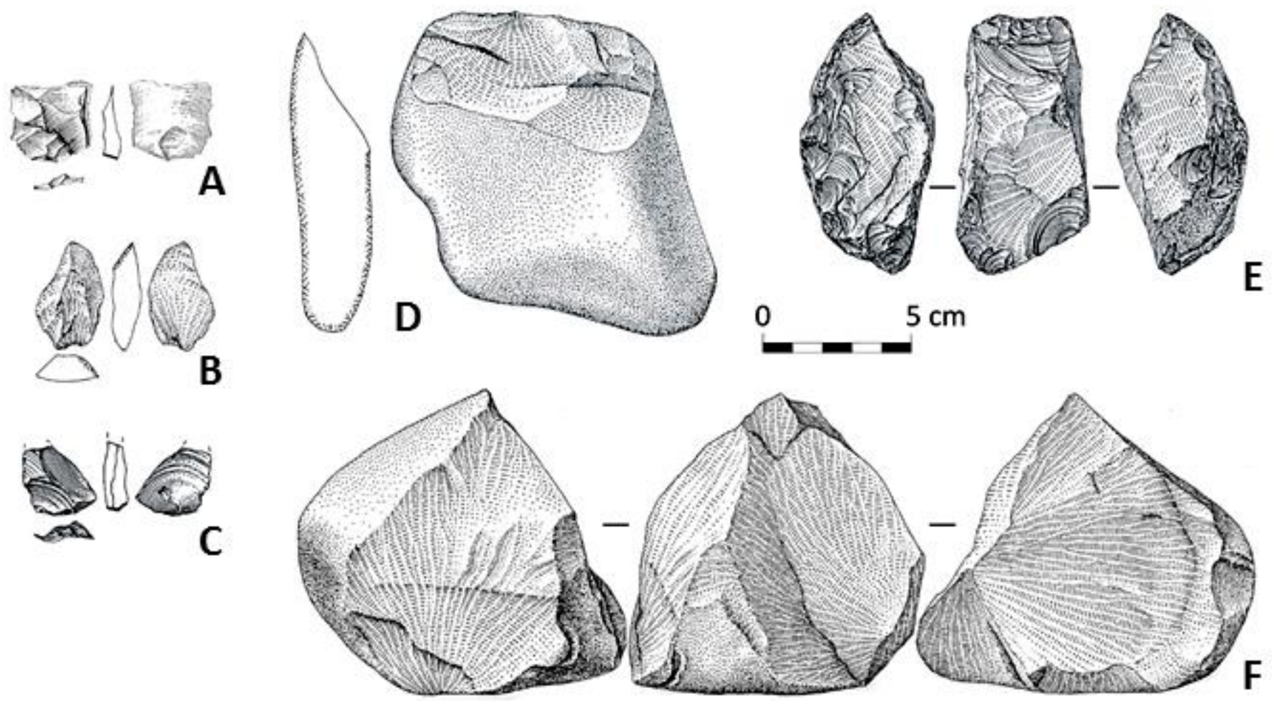

Supplementary Fig. 4. Lithic industry of Vallonnet Cave (refs. 74-76). **(A)**, **(B)**, **(C)** Flakes. **(D)** Chopper. **(E)** Core, with unifacial, bipolar removals, obtained from two natural cortical striking platforms. **(F)** Core with multidirectional, alternate, perpendicular removals, on thick pebble.

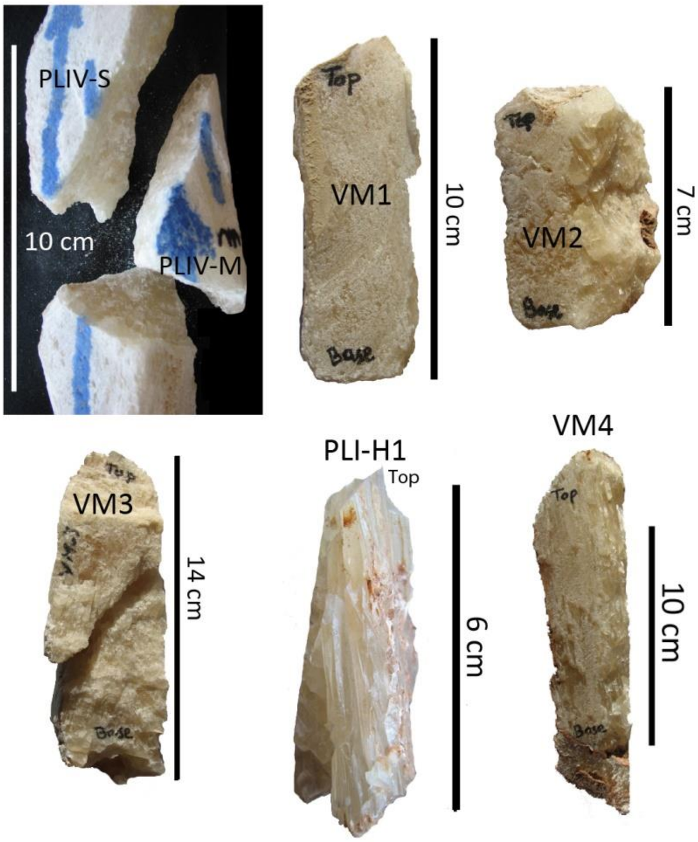

Supplementary Fig. 5. **Vallonnet Cave samples of well-crystallized calcite for U-Th and U-Pb analyses**. Upper flowstone of complex IV (PLIV-S, PLIV-M, VM1, VM2, VM3) and Lower flowstone of complex I (PLI-H1, VM4).

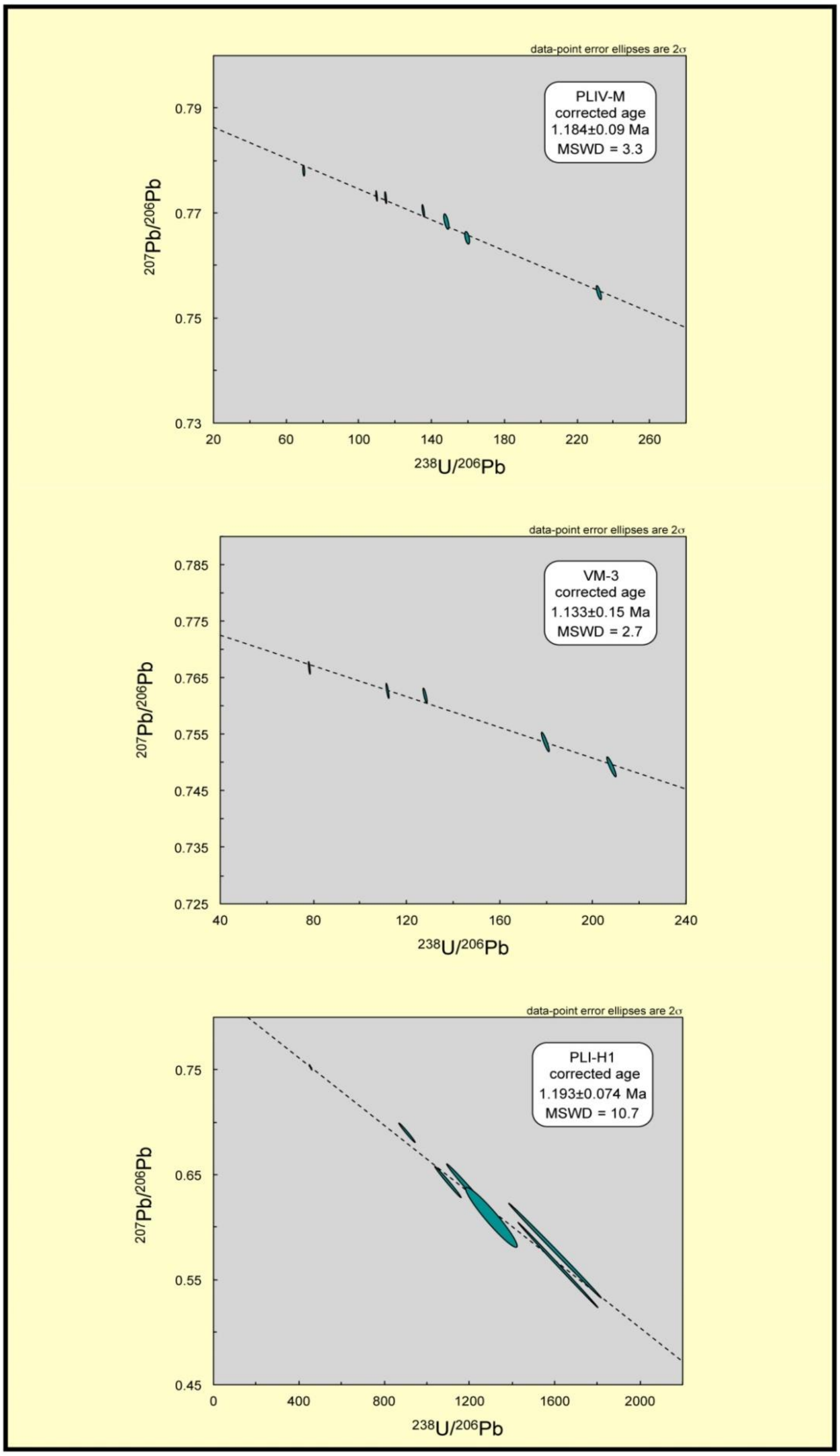

Supplementary Fig. 6. U-Pb age data plotted in the Tera-Wasserburg isochron construction[77] with ages corrected for initial disequilibrium in the U-series decay chain.

Supplementary Table 1. **Taxa list of archaeological deposits at complex III, in Vallonnet Cave.**

Macaca sylvanus florentina (ref. 24)
Ursus deningeri (ref. 24)
Xenocyon lycaonoides (ref. 78)
Canis mosbachensis (ref. 24)
Vulpes praeglacialis (ref. 24)
Pachycrocuta brevirostris (ref. 24)
Homotherium crenatidens (ref. 79)
Acinonyx pardinensis (ref. 80)
Panthera gombaszoegensis (ref. 24)
Panthera pardus (ref. 24)
Lynx spelaeus (ref. 24)
Felis silvestris (ref. 24)
Meles meles (ref. 24)
Mammuthus meridionalis (ref. 80)
Stephanorhinus hundsheimensis (ref. 81)
Equus stenonis (ref. 80)
Hippopotamus cf. *antiquus* (ref. 82)
Sus sp. (ref. 80)
Bison schoetensacki (ref. 79)
Praeovibos sp. (ref. 79)
Ammotragus europaeus (ref. 55)
Hemitragus bonali (ref. 24)
Rupicaprini gen. ind. sp. ind. (ref. 79)
Praemegaceros cf. *verticornis* (ref. 79)
Pseudodama nestii vallonnetensis (ref. 24)
Oryctolagus gr. *laynensis-cuniculus* (ref. 83)
Hystrix refossa (ref. 80)
Ungaromys nanus (ref. 84)
Mimomys aff. *savini* (ref. 84)
Microtus (*Allophaiomys*) cf. *nutiensis* (ref. 84)
Microtus (*Iberomys*) ex gr. *huescarensis-brecciensis* (ref. 84)
Pliomys cf. *lenki* (ref. 84)
Beremendia fissidens (ref. 84)
Testudo hermanni (ref. 85)
Malpolon monspessulanus (ref. 85)

Supplementary Table 2. **Uranium and thorium isotopic compositions of Vallonnet Cave samples by MC-ICP-MS at HISPEC, NTU.**

Sample ID	Levels	^{238}U (ng/g)		^{232}Th (pg/g)		$[^{230}Th/^{232}Th]$ activity		$[^{234}U/^{238}U]$ activity	
PLIV-S	IV top	314.56	± 0.29	213.5	± 0.9	5232	± 22	1.0875	± 0.0013
PLIV-Sb[1]		348.09	± 0.32	319.2	± 0.6	4056	± 9	1.1145	± 0.0014
VM1	IV top	301.44	± 0.33	31.8	± 10.6	29879	± 9945	1.0236	± 0.0015
VM1b[1]		364.79	± 0.38	62.0	± 7.2	18491	± 2138	1.0203	± 0.0014
PLIV-M	IV middle	315.30	± 0.39	60.1	± 0.4	16469	± 122	1.0242	± 0.0024[2]
PLIV-Mb[1]		305.83	± 0.30	47.2	± 0.4	20345	± 192	1.0225	± 0.0013[2]
VM2	IV base	345.59	± 2.71	193.9	± 4.6	5627	± 136	1.0514	± 0.0089
VM2b[1]		236.19	± 0.24	32926.7	± 334.3	26.5	± 0.4	1.4519	± 0.0017
VM3	IV base	533.49	± 0.50	274.2	± 8.6	6099	± 191	1.0203	± 0.0013[2]
VM3b[1]		442.80	± 0.56	66.2	± 5.2	20937	± 1641	1.0194	± 0.0014[2]
PLI-H1	I top	285.16	± 0.35	580.4	± 0.9	1539	± 4	1.0200	± 0.0025[2]
PLI-H1b[1]		2208.69	± 2.26	1308.5	± 1.4	5297	± 9	1.0231	± 0.0014[2]
VM4	I top	173.56	± 0.22	125.8	± 5.7	4329	± 198	1.0200	± 0.0016
VM4b[1]		212.39	± 0.31	130.8	± 8.0	5078	± 311	1.0173	± 0.0021

[1] Duplicate.
[2] Values used to calculate U-Pb isochron ages corrected for initial $^{234}U/^{238}U$ disequilibrium (see Supplementary Table 3).
Analytical errors are 2σ of the mean.

Supplementary Table 3. **Uranium and lead isotopic compositions of Vallonnet Cave samples by MC-ICP-MS, The University of Melbourne.**

Complex	Sample ID	U ppb	Pb ppb	$^{238}U/^{206}Pb$	% err	$^{207}Pb/^{206}Pb$	% err	corr. coef.	$^{234}U/^{238}U$ * measured	Corrected age (Ma)
IV middle	PLIV-M	307.82	4.22	231.51	0.49	0.7548	0.14	-0.758	1.0225 ± 0.0013	1.18 ± 0.09
		340.54	10.08	108.92	0.31	0.7733	0.10	-0.692	1.0242 ± 0.0024	1.17 ± 0.09
		322.03	7.71	134.54	0.42	0.7705	0.12	-0.726		
		303.42	8.58	114.03	0.35	0.7729	0.12	-0.642		
		327.40	6.61	158.82	0.67	0.7653	0.13	-0.517		
		322.17	7.04	147.35	0.81	0.7684	0.16	-0.660		
		347.92	16.39	68.77	0.57	0.7781	0.11	-0.306		
IV base	VM3	532.90	9.44	179.53	0.76	0.7536	0.19	-0.901	1.0203 ± 0.0013	1.13 ± 0.15
		405.46	16.71	77.84	0.36	0.7667	0.12	-0.736	1.0194 ± 0.0014	1.14 ± 0.15
		404.48	6.15	208.11	0.76	0.7493	0.19	-0.906		
		383.58	11.01	111.56	0.54	0.7627	0.14	-0.824		
		424.15	10.62	127.69	0.53	0.7618	0.14	-0.823		
I top	PLI-H1	253.19	0.64	1213.51	8.45	0.6317	3.72	-0.995	1.0200 ± 0.0025	1.22 ± 0.09
		214.50	0.49	1299.18	7.58	0.6095	3.84	-0.955	1.0231 ± 0.0014	1.19 ± 0.07
		179.47	0.32	1611.29	9.57	0.5641	5.86	-0.999		
		243.82	1.75	447.50	1.33	0.7524	0.29	-0.954		
		241.15	0.65	1093.93	4.57	0.6432	1.88	-0.992		
		278.48	0.52	1596.31	11.14	0.5779	6.38	-0.999		
		366.66	1.15	901.54	3.48	0.6899	1.10	-0.986		

Uncertainties on isotope ratios are quoted at 2σ. Age uncertainties are 95% confidence.
Isochron-derived ages are corrected for initial disequilibrium in the U series decay chain using the measured $^{234}U/^{238}U$ values noted above.
^{234}U and U-Pb measurements were performed on different sample aliquots and thus ages are calculated separately for each ^{234}U determination. In all cases the resulting age determinations are within uncertainty of each other.
* from Table S2.

Supplementary Table 4. **Paleomagnetism data of Vallonnet Cave deposits.**

Complex	Deposit	Sample ID	Intensity (A/m)	Inclination (°)	Declination (°)
V	Colluviums	PM3-1	1.10E-06	51.1	287.69
		PM3-2	1.09E-06	77.41	223.32
		PM3-3	6.62E-06	15.03	350.53
IV	Upper Flowstone	PM5-1	4.79E-08	-22.08	172.14
		PM5-2	2.27E-08	-34.78	255.97
		PM5-3	8.60E-08	-12.15	241.15
III	Archaeological levels: sand/gravel	PM5-4	1.49E-07	-7.71	226.26
		PM1-1	2.66E-06	70.18	12.92
		PM1-2	5.99E-06	70.11	41.85
		PM1-3	5.07E-06	58.31	15.71
		PM2-1	1.98E-06	38.17	7.93
		PM2-2	1.54E-06	59.82	4.31
		PM2-3	1.86E-06	53.11	356.26
		PM2-4	1.48E-06	70.83	168.06
		PM2-5	1.48E-06	56.15	2.16
II	Beach	not measured			
I	Lower Flowstone	PM4-1	5.18E-08	-48.17	295.78
		PM4-2	1.83E-08	-16.44	293.72

Supplementary Note 1. Stratigraphy and paleoenvironment

Vallonnet Cave is located 110 m above the present-day sea level at Roquebrune-Cap-Martin in France (Supplementary Fig. 1A and 1B). The cave is carved in a dolomitic limestone massif from the Jurassic period, pitched, enveloped in a Miocene conglomerate made up of pebbles and sandy concretions[74]. Inside the cave, abundant fossil mammal remains and artefacts were recovered during multiple excavations conducted since 1962 (Supplementary Fig. 1C and 1D). Five stratigraphic complexes (I-V), corresponding to five climatic periods, have been identified in the 2 m Quaternary infilling of the cave (Fig. 4 and Supplementary Fig. 2) (refs. 27, 74, 86).

Complex I corresponds to a warm and humid climate. The landscape was covered with large deciduous trees such as *Platanus* and *Pterocarya*, Mediterranean taxa and *Pinus*[48]. In these climatic conditions, a flowstone, 25 cm in thickness, formed in the cave at the base of the infilling, directly on the limestone bedrock. Only strips of this substratum remain along the walls of the cave entrance and at the base of the sedimentary deposits in the main chamber. This lower flowstone contains pure, well-crystallized and compact calcite. It was substantially eroded after its formation, during a period of rising sea levels.

Complex II corresponds to a clearly warm climate with thermophilous tree cover, dominated by pine groves and Mediterranean taxa[48]. As the sea level rose following a eustatic transgression linked to the melting of glaciers, Vallonnet Cave became a coastal site and was inhabited by marine organisms. The corresponding marine deposits are represented mainly by sands rich in marine fauna suggestive of tropical seas[87]. During this period, the sea profoundly sculpted the rock and the flowstone of complex I, and led to the dismantling of the continental infilling, leaving only strips of stalagmites. In addition, marine molluscs perforated the cave walls.

Complex III corresponds to climatic cooling and a marine regression. Sands, clays and silts were deposited in the cave by run-off (Fig. 4). These sediments contained a rich land vertebrate fauna and lithic industries, attesting to early hominin presence in the southeast of France. Complex III was deposited during a relative cool and dry period but with sufficiently long and warm summers for the reproduction of Hermann's tortoise and the Montpellier snake[27]. This evidence of cold climate is supported by the graphical representation of the mammalian community structure[88]. The lower deposits, called C and B2, correspond to a level of blocks and stones with a reddish-yellow clayey-sandy matrix, about 40 cm thick. These deposits correspond to a cold, very dry climate with a steppe-type landscape, where the trees were decimated by the drop in temperature[48]. Several archaeostratigraphic units, with sub-horizontal deposits, were identified. At the top of the sequence, the deposits, called B1, are made up of heterogeneous stones with a reddish-yellow clayey-sandy matrix reaching a thickness of nearly 90 cm in places. The coarse elements comprise limestone fragments, pebbles and calcite fragments with strong alteration of coarse elements in some sectors of the cave. The presence of pebbles from the Miocene pudding stone (situated above the Jurassic limestone massif in which Vallonnet Cave is carved) provides evidence of intense periodic run-off events. These upper deposits formed under temperate and humid climatic conditions with a slight increase in tree cover, mixed with oak and pine grove and Mediterranean taxa[27,48]. Five archaeostratigraphic units with sub-horizontal deposits were identified.

Complex IV is a thick flowstone (upper flowstone) that formed above complex III, and sealed the corridor of the cave entrance after climatic warming, preserving the remains from further erosion. It formed during an interglacial period in a forested landscape, with dominant pine groves, Mediterranean species and large deciduous trees. The flowstone was partly removed to access the cave infilling for excavations. It is presently only conserved in the west of the site, against the cave wall, where it is 40 cm thick. This flowstone precipitated with well-crystallized, pure and compact calcite.

Later, the upper Complex V are colluviums (without faunal remains and lithic industry) deposited from run offs with older sediments during wet Quaternary phases. Colluviums deposited

after the flowstone of complex IV. They are made up of pebbles in a yellowish-red sandy matrix and seal the continental infilling of complex III.

It is shown with the well-documented descriptive caption that the Stalagmitic Floor (Complex IV with reverse paleomagnetic polarity) formed after Complex III (Archaeological levels with normal paleomagnetic polarity) (Figure 4, Supplementary Figure 2). The sediments in Complex III deposited during the presence of faunal mammals and hominins in the cave when the entrance of the cave opened (Supplementary Fig 1A). The stalagmitic floor (Complex IV) then formed and definitively closed the entrance of the cave, preserving the remains from further erosion. Later, the upper deposits from Complex V are colluviums (without faunal remains and lithic industry) from older deposits during wet Quaternary phases. In addition, at the beginning of the excavations, the stalagmitic floor covering Complex III had to be partially destroyed to access the cave entrance, as described in Supplementary Figure 2. Accordingly, Complex IV is definitely younger than Complex III and it represents a clear minimum age for Complex III.

In summary, we studied the stratigraphy of the site over the past 20 years and the geological history of the infill is well established. Supplementary Figure 2 shows that the site is absolutely not problematic with respect to the stratigraphy of Complexes III, IV and V: Complex III (archaeological levels) pre-dates the formation of the Complex IV (Stalagmitic floor) and Complex V (colluviums) post-dates the Complex IV. The Complex IV post-dates Complex III because it definitely closed the entrance of the cave, preserving the archaeological levels (Complex III) from further erosion, then colluviums (Complex V without archaeological material) were deposited from run offs during wet periods. The first phase of fieldwork on the site required breaking the stalagmitic floor covering the sediment infilling (Complex III) which is a clear evidence of the Complex IV posteriority against the Complex III.

Supplementary Note 2. The lithic assemblage

The lithic series is composed of 97 artefacts, most of which were discovered in the complex III levels (C, B2 and B1), mixed with bones[28]. The small lithic series has been questioned in the past[29,30] but numerous studies on both the technologies applied and the raw materials indicate a clear anthropic origin, remains of a scarce and sporadic occupation by hominin of the cave for bivouacs. The assemblage is made from local limestone pebbles from a Miocene conglomerate, and includes some objects in quartzite, sandstone and flint (Fig. 3 and Supplementary Fig. 4). Considering the topography and features of the limestone cave, these objects could not have been introduced into the cave by natural processes, due to their size. Moreover flint pebbles were collected from 700 m north of the cave at Ciotti, near Menton city[89,74]. The series is mainly composed of pebbles used as percussion instruments (showing isolated convex removals and/or percussion marks), pebble-tools, unretouched flakes and two cores[28]. *Débitage* flakes total 11 items, including two in flint (Fig. 3C and 3D). The flakes indicate clear technical features of intentional knapping (striking platform, bulb, and structured organization of the removals on the upper face) (Supplementary Figs. 4A, 4B, 4C). One core is made on a thick limestone pebble without percussion marks and presents multidirectional surfaces with large and short removals (Fig. 4F). The second core is in flint (Fig. 3E, Supplementary Fig. 4E). Removals are bipolar from two cortical platforms. Percussion marks are visible on some cutting edges. This technique of debitage is described in several Early Pleistocene European sites and reinforces the anthropic origin of the lithic series found at Vallonnet Cave. This technic is applied on various types of stones, flint and quartz for instance, indicating the diversity of skills of Mode 1 hominins and their ability to adapt their behavior to specific stone shapes and sizes. We may mention the sites of

Vallparadis, Untermassfeld, Pont-de-Lavaud or Orce (refs. 90-95, 14, 96-98). The refitting of some pebble-tools with structured removals (Supplementary Fig. 4D) and pebbles with isolated removals[28,76] (Fig. 3H) attests to *in situ* knapping and percussion activities in the cave to reshape the tools and probably to break the bones. The absence of core refitting suggests that flakes were brought from outside the cave. The corpus belongs to the core-and-flake industry type, commonly found in other series from European sites around 0.9-1.5 Ma (refs. 7, 8, 10, 13-17, 44, 91, 99-100). The composition of the series and the technical features correspond to a tool kit conducive to scavenging the remains of carcasses left by carnivores in a small den.

Supplementary Note 3. Fauna, biostratigraphy and taphonomy

The taxonomic identification of the large mammalian species from complex III indicates that they belong to the Epivillafranchian biochron[24,25] (see Fig. 2 and Supplementary Table 1). Moreover micromammal assemblages include characteristic Early Pleistocene taxa[84]. The Vallonnet micromammal list contains *Beremendia fissidens*, *Ungaromys nanus*, *Mimomys* aff. *savini*, *Microtus (Allophaiomys)* cf. *nutiensis*, *Microtus (Iberomys)* such as gr. *huescarensis-brecciensis* and *Pliomys* cf. *lenki*[84]. The micromammals *Beremendia fissidens*, *Allophaiomys nutiensis*, *Ungaromys* cf. *nanus* are reported[101] in the TELRU Levels of la Sima del Elefante at Atapuerca, mainly in levels TE7 and TE9, dated respectively to 1.13 ± 0.18 Ma and 1.22 ± 0.16 Ma (ref. 9). *Ungaromys nanus* is also reported at Vallparadis level 10 (after Jaramillo)[16]. *Allophaiomys* aff. *nutiensis* was determined at Bois-de-Riquet (BDR 1) in a level with an estimated age of 1.2 Ma (ref. 10). *Mimomys savini* is present at Atapuerca starting from level TD 3-4 (Gran Dolina)[101], ~0.9 Ma (ref. 102), at Untermassfeld[103], at Fuente Nueva-3 (ref. 104) and at Vallparadís level 10 (after Jaramillo)[105,16]. *Pliomys lenki* is present at Colle Curti[106]. The micromammal faunal association from Vallonnet, in particular rodent species and their evolution stage[107], is consistent with an age of 1.2 Ma.

A previous taphonomic study on large mammals was carried out[26]. The surface of each bone (identified or not identified) was carefully examined with hand lenses to isolate all the damages[26].The remains are highly fragmented due to pressure from overlying sediments and trampling. The cave records alternating carnivore and hominin occupations. Bears died in cave, which was also used as a den by carnivores, in particular by hyenas. Porcupines also visited the cave and left marks on fresh bones.

Many bones and some of them with anthropic or carnivore marks brought to the cave by hominins and/or by carnivores were found among these deposits. Anthropic marks were identified on twelve faunal bones. Only five remains are related to Bison (one metatarsal and one femur), *Praemegaceros* (one humerus), *small ungulate* (one vertebra), *Pseudodama* (one tibia) (ref. 26). On the *Praemegaceros* humerus (Supplementary Fig. 3A), a series of marks of different width was observed on the lateral epicondylar crest (Supplementary Fig. 3B). Their trajectories are straight and deep with an oblique orientation to the bone axis. The microscopic (SEM) analysis shows a V-shaped in cross-section (Supplementary Fig. 3C) with internal straight microstriations (Supplementary Fig. 3D). All these criteria indicate diagnostic cut-marks related to butchery activities. Their location arises from cut muscular tendon (filleting) by using stone tool[108]. In Vallonnet Cave, all the cut-marks correspond to removal meat probably due to a scavenging behavior as it is observed at Bois-de-Riquet[10]. At Untermassfeld site, a predominance of cut-marked specimens from lower limbs indicate that hominins had late access to megaherbivore carcasses (rhinoceros and hippopotamus)[109]. Whereas, different butchery activities (skinning, dismembering, defleshing) indicate a primary and early access to the animals consumed by hominins, as observed in the groups of small-sized and medium-size mammals from Untermassfeld and on some ungulate remains from la Sima del Elefante (Level TE9c)[46].

Materials and Methods for taphonomic analysis

Microscopic analysis of cut-marks was carried out on a left humerus of *Praemegaceros* (Supplementary Fig. 3). High-resolution replicas were made using Silicon (President Microsytem). Positive replicas were then made using an Epoxy Resin. These replicas were coated with platinum and analysed using a Tescan Vega3 XM SEM (Scanning Electron Microscope) at a 5 kV accelerating voltage. Several criteria have been used to identify butchery cut-marks from marks imparted by non-anthropic agents, such as carnivores, rodents or trampling marks: anatomic location of the marks, orientation to the bone axis, macro and micro-morphology[108,110,111].

Supplementary References

73. Lumley, H. de. La grotte du Vallonnet, Roquebrune-Cap-Martin, Alpes-Maritimes, Situation géographique, Description, Historique. *Anthropologie* **92**, 387-397 (1988).

74. Lumley, H. de, Barsky, D. & Cauche, D. Les premières étapes de la colonisation de l'Europe et l'arrivée de l'Homme sur les rives de la Méditerranée. *Anthropologie* **113**, 1-46 (2009).

75. Lumley de H., Barsky D. and Cauche D. Archaic Stone Industries from East Africa and Southern Europe Pre-Oldowan and Oldowan. (*The Cutting Edge: New approaches to the Archaeology of Human Origins*, Eds. K. Schick and N. Toth), 3, Chapter 4, 55-92 (2009).

76. Lumley, H. de, Fournier, A., Krzepkowska, J. & Echassoux, A. L'industrie du Pléistocène inférieur de la grotte du Vallonnet. Roquebune-Cap-Martin, Alpes-Maritimes. *Anthropologie* **92**, 501-614 (1988).

77. Ludwig, K.R. *User's manual for Isoplot 3.0. A Geochronological Toolkit for Microsoft Excel* (Berkeley Geochronology Center, Special Publication, 2003).

78. Moullé, P.-E. & Echassoux, A. Étude du Xenocyon lycaonoides de la grotte du Vallonnet (Roquebrune-Cap-Martin, Alpes-Maritimes). *Bull. Mus. Anthropol. Préhist. Monaco* **45**, 15-24 (2005).

79. Moullé, P.-E. Les grands mammifères de la grotte du Vallonnet (Roquebrune-Cap-Martín, Alpes Maritimes). Synthèse des études antérieures et nouvelles déterminations. *Bull. Mus. Anthropol. Préhist. Monaco* **39**, 29-36 (1998).

80. Lumley, H. de, Gagnière, S., Barral, L. & Pascal, R. La grotte du Vallonnet (A.-M.). *Bull. Mus. Anthropol. Préhist. Monaco* **10**, 5-20 (1963).

81. Lacombat, F. *Les rhinocéros fossiles des sites préhistoriques de l'Europe méditerranéenne et du Massif Central. Paléontologie et implications biochronologiques*. Oxford, BAR International Series 1419 (2005). ISBN: 9781841718583

82. Moullé, P.-E., Echassoux, A., Lacombat, F., Desclaux, E. & Bailon, S. L'environnement animal des premiers habitants de l'Europe méditerranéenne : les grands mammifères contemporains de l'Homme du Vallonnet, données taxonomiques et biostratigraphiques pour la deuxième moitié du Pléistocène inférieur. *Les premiers peuplements en Europe: Données récentes sur les modalités de peuplements et sur le cadre chronostratigraphique, géologique et paléogéographique des industries du Paléolithique ancien et moyen en Europe*, eds Molines N, Moncel M-H, Monnier JL. Oxford, BAR International Series **1364**, 105-113 (2005).

83. Nocchi, G. & Sala, B. The fossil rabbit from Valdemino Cave (Borgio Verezzi, Savona) in the context of western Europe Oryctolagini of Quaternary. *Palaeovertebrata*, Montpellier, **26**, 167-187 (1997).

84. Desclaux, E. Évolution des communautés de mammifères dans les Alpes-Maritimes au cours du Pléistocène. *Bull. Mus. Anthropol. Préhist. Monaco* **4,** 49-59 (2013).

85. Bailon, S. & Blain, H.A. Faunes de reptiles et changements climatiques en Europe occidentale autour de la limite plio-pléistocène. *Quaternaire* **18**, 55-63 (2007).

86. Lumley, H. de. La stratigraphie du remplissage de la grotte du Vallonnet. *Anthropologie* **92**, 407-428 (1988).

87. Granier, J. La Faune marine fossile des dépôts inférieurs de la grotte du Vallonnet, Roquebrune-Cap-Martin (Alpes-Maritimes). *Anthropologie* **92**, 449-460 (1988).

88. Montuire, S. & Desclaux, E. Analyse paléoécologique des faunes de mammifères et évolution des environnements dans le sud de la France au cours du Pléistocène. *Quaternaire* **8**, 13-20 (1997).

89. Grégoire, S. Nature des roches et aires d'approvisionnement en matière première des industries lithiques archaïques des premiers habitants de l'Europe. Exploitation des territoires. *Anthropologie* **113**, 168-177 (2009).

90. Barsky, D. *et al.* Raw material discernment and technological aspects of the Barranco León and Fuente Nueva 3 stone assemblages (Orce southern Spain). *Quat. Int*. **223-224**, 201–219 (2010).

91. de Lombera-Hermida, A. *et al.* The lithic assemblage from Pont-de-Lavaud (Indre, France) and the role of the bipolar-on-anvil technique in the Lower and Early Middle Pleistocene technology. *J. Anthropol. Archaeol.* **41**, 59-184 (2016).

92. Garcia, J., Martinez, K., Carbonell, E. The Early Pleistocene stone tools from Vallparadis (Barcelona, Spain): Rethinking the European Mode 1. *Quat. Int.* **316**, 94-114 (2013).

93. Garcia, J., Landeck, G., Martínez, K., Carbonell, E. Hominin dispersals from the Jaramillo subchron in central and south-western Europe: Untermassfeld (Germany) and Vallparadís (Spain). *Quat. Int.* **316**, 73-93 (2013).

94. Hiscock, P. Dynamics of knapping with bipolar techniques: modelling transitions and the implications of variability. *Lithic Technology* **40**, 342-348 (2015).

95. De la Peña, P. A qualitative guide to recognize bipolar knapping for flint and quartz. *Lithic Technology* **40**, 316-331 (2015).

96. Martinez, K. *et al.* A new Lower Pleistocene archaeological site in Europe (Vallparadis, Barcelona, Spain). *P. Natl. Acad. Sci. USA* **107**, 5762-5767 (2010).

97. Stout, D. & Semaw, S. Knapping skill of the earliest toolmakers: insights from the study of modern human novices (*The Oldowan: Case Studies into the Earliest Stone Age*, Eds. Toth, N., Schick, K). Stone Age Institute Press, Gosport, chapter 10, 307-320 (2006).

98. Vergès, J.M., Ollé, A., 2011. Technical MicroWear and Residues in Indentifying Bipolar Knapping on an Anvil : Experimental Data. *J. Archaeol. Sci.* **38**, 1016-1025 (2011).

99. Espigares, M.P. *et al. Homo vs. Pachycrocuta*: Earliest evidence of competition for an elephant carcass between scavengers at Fuente Nueva-3 (Orce, Spain). *Quat. Int.* **295**, 113-125 (2013).

100. de Lombera-Hermida, A. *et al.* The lithic industry of Sima del Elefante (Atapuerca, Burgos, Spain) in the context of Early and Middle Pleistocene technology in Europe. *J. Hum. Evol.* **82**, 95-106 (2015).

101. Cuenca-Bescos, G. *et al.* Updated Atapuerca biostratigraphy: Small-mammal distribution and its implications for the biochronology of the Quaternary in Spain. *C. R. Palevol* **15**, 621-634 (2016).

102. Rodriguez J. *et al.* One million years of cultural evolution in a stable environment at Atapuerca (Burgos, Spain). *Quat. Sci. Rev.* **30**, 1396-1412 (2011).

103. Maul, L. Die Kleinsäugerreste (Insectivora, Lagomorpha, Rodentia) aus dem Unterpleistozän von Untermaßfeld. *Das Pleistozän von Untermaßfeld bei Meiningen (Thüringen), Teil 3*, eds. Kahlke RD (Bonn, Monographien des Römisch-Germanischen Zentralmuseums Mainz) 40, pp. 783-887 (2001).

104. Agusti, J., Blain, H.-A., Furio, M., De Marfa, R. & Santos-Cubedo, A. The early Pleistocene small vertebrate succession from the Orce region (Guadix-Baza Basin, SE Spain) and its bearing on the first human occupation of Europe. *Quat. Int.* **223-224**, 162-169 (2010).

105. Garcia, J., Martínez, K., Cuenca-Bescos, G., Carbonell, E. Human occupation of Iberia prior to the Jaramillo magnetochron (>1.07 Myr). *Quat. Sci. Rev.* **98**, 84-99 (2014).

106. Abbazzi, L., Masini, F., Ficcarelli, G. & Torre, D. Arvicolid finds (Rodentia, Mammalia) from the early Galerian of Colle Curti (Umbro-Marchean Apennines, Central Italy). *Acta zool. Cracov*. **41**, 133-142 (1998).

107. Maul L. C., Heinrich W.-D., Parfitt S. A., Paunescu A.-C. Comment on the correlation between magnetostratigraphy and the evolution of Microtus (Arvicolidae, Rodentia, Mammalia) during the Early and early Middle Pleistocene. *Cour. Forsch.-Inst. Senckenberg* **259**, 243-263 (2007).

108. Fernandez-Jalvo, Y., Andrews, P. Atlas of Taphonomic Identifications (1001+Images of Fossil and Recent Mammal Bone Modification). Vertabrate Paleobiology and Paleoanthropology Series, Springer Dordrecht (2016). ISBN: 978-94-017-7430-7

109. Landeck, G., Garcia Garriga, J. New taphonomic data of the 1 Myr hominin butchery at Untermassfeld (Thuringia, Germany). *Quat. Int.* **436**, 138-161 (2017).

110. White, T.D. *Prehistoric cannibalism at Mancos 5MTUMR-2346*. Princeton Univ. Press, Princeton, NJ. (1992). ISBN: 0-691-08539-0

111. Defleur, A., White, T., Valensi, P., Slimak, L., Crégut-Bonnoure, E. Neanderthal cannibalism at Moula-Guercy, Ardèche, France. *Science* **286**, 128-131 (1999).
